# *hackseq*: Catalyzing collaboration between biological and computational scientists via hackathon

**DOI:** 10.12688/f1000research.10964.2

**Published:** 2017-04-10

**Authors:** hackseq Organizing Committee 2016

**Keywords:** Hackathon, Genomics, Bioinformatics, Open Science, Diversity in Science

## Abstract

*hackseq* ( http://www.hackseq.com) was a genomics hackathon with the aim of bringing together a diverse set of biological and computational scientists to work on collaborative bioinformatics projects. In October 2016, 66 participants from nine nations came together for three days for *hackseq* and collaborated on nine projects ranging from data visualization to algorithm development. The response from participants was overwhelmingly positive with 100% (n = 54) of survey respondents saying they would like to participate in future hackathons. We detail key steps for others interested in organizing a successful hackathon and report excerpts from each project.

## Introduction

Technological advances in the biological sciences have led to an increasing availability of so-called ‘-omic’ datasets, allowing fundamental questions in biology to be answered at an unprecedented rate ^1^. However, these datasets are complex, requiring novel and specialized informatics tools for proper analysis and to overcome the computational bottleneck in research. Open-source bioinformatics tools and pipeline development accelerates the rate of research by allowing the community to both reuse and thoroughly assess such methods. Thus, by solving biological problems in an open and collaborative manner, the field can progress at a faster rate than if code remains unavailable to the larger community ^2^.

Hackathons offer a solution to catalyze tool and pipeline development for biological data science, as well as foster interdisciplinary collaborations ^3^. These events aim to solve defined computational problems over a period of days by bringing together small teams of individuals with different and diverse skillsets. Although frequently valuable for the outputs they generate, hackathons have faced criticism due to low levels of diversity amongst participants ^4^. We therefore established *hackseq*, a genomics hackathon collective ( http://www.hackseq.com) that aims to promote open science, collaboration and diversity. We placed special emphasis in promoting leadership amongst women, minorities and early-career scientists. The inaugural *hackseq* event took place over three days in October 2016 in Vancouver (British Columbia, Canada) and was a satellite event to the annual American Society of Human Genetics (ASHG) meeting. Here we report a summary of this hackathon in the hopes of promoting similar events in the future.

## *hackseq* format

*hackseq* was the first genomics hackathon in Vancouver and was based on the NCBI hackathon format ^3^. Some hackathons can be perceived as high-pressure events exclusive to technically inclined and experienced individuals. We thus took measures to ensure that people of all skill levels and backgrounds were encouraged to apply. We structured *hackseq* as a three-day event that runs primarily from 8AM – 5PM on the Saturday/Sunday/Monday prior to the 2016 ASHG meeting. The *hackseq* itinerary is accessible on the *hackseq* github repository ( https://github.com/hackseq/October_2016/blob/master/hackseq_2016_schedule.md).

First, we opened a call for ‘team leaders’ to propose a project and lead a small team at *hackseq* through social media, such as Twitter, making announcements at the Vancouver Bioinformatics User Group (VanBUG) and bioinformatics.ca, as well as direct email contact with potential leaders. We screened the projects to confirm that their aims and scope would be appropriate for a 72 hour hackathon. For the ten accepted projects we used GitHub as a discussion board, creating issue threads ( https://github.com/hackseq/hackseq_projects_2016/issues) for each project, allowing prospective participants to view and discuss the details about each project before applying to join a particular team.

Once we established the ten *hackseq* projects, we opened the call for participants. Our main goal in recruiting participants was to reach out to a diverse group of individuals and to promote participation of women, minorities and early-career scientists. To this end, we partnered with organizations, such as Society for Canadian Women in Science and Technology (SCWIST) and VanBUG, to attract local participants. To encourage early-career scientist involvement, we contacted undergraduate and graduate-level computational sciences and bioinformatics programs at regional universities. To reach the global scientific audience, we contacted several human genetics societies around the world asking them to email participant application information to their respective mailing lists.

To promote economic diversity and lower the barrier to entry for international participants, we partnered with ASHG to create travel awards based on financial need and/or minority status. *hackseq* had no registration fee. Lastly, we made announcements on Twitter, Galaxy Project’s events calendar, and various international conferences, such as Bioinformatics Open Source Conference 2016 and the 13 ^th^ International Congress of Human Genetics, leading up to the hackathon.

In the participant application form, prospective participants ranked the top four projects on which they would like to work. Participants could apply for travel awards by ASHG and request child care, covered by our budget, to promote participation amongst parents. The organizing committee and the team leaders evaluated the applications based on not only their skill levels, but also their interests and passion for genomics. To ensure well-rounded teams, we considered both the project preferences and skill levels during the team assignment phase, ensuring a balance between novices and expert coders, and biological and computational expertise. All forms developed for *hackseq* are available online ( https://github.com/hackseq/October_2016/blob/master/Forms.md).

By defining the projects and teams beforehand, participants got to know their team members and prepare technical infrastructure. Teams hit the ground running on the first day, beginning work unprompted by the organizers at 8AM of the first day.

**Figure 1.**
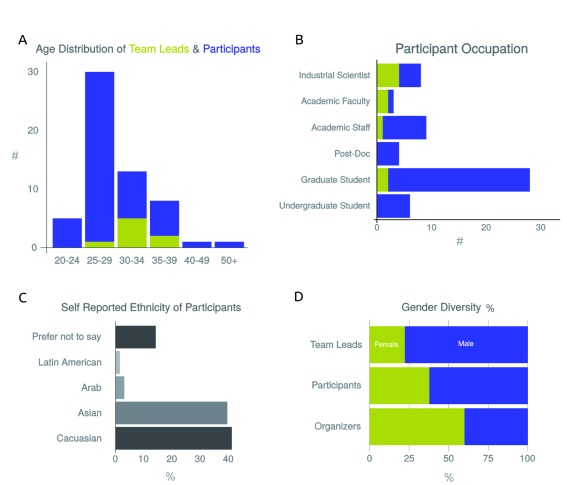
Participant diversity at hackseq 16. To measure diversity of hackseq participants, we asked team leaders and participants to self-report their ( **A**) age, ( **B**) current occupation, ( **C**) ethnicity and ( **D**) gender. Data is shown for team leaders (yellow) and participants (blue).

*hackseq* had 66 participants in attendance from nine nations, divided into nine teams ranging from 3 to 10 individuals. Of the accepted ten projects, two team leaders withdrew prior to the hackathon for personal reasons, and one popular project split into two teams, resulting in nine teams. The mode age-category was 30–34 years old (62.5%) for team leaders, and 25–29 years old (58%) for participants ( Figure 1A). Graduate students made up the largest fraction of participants with 48.2%, followed by academic staff (15.5%), industrial scientists (13.8%), undergraduates (10.3%), postdoctoral fellows (6.9%) and academic faculty (5.2%). Notably, the team leaders were more likely to be industry scientists (44.4%) or young faculty (22.2%) ( Figure 1B). In total, 22 of 62 (35.5%) participants identified as female and 40 as male. A total of 41% self-identified as Caucasian, 40% as Asian or Pacific Islander, and 19% as Arab, Latin American or unspecified ( Figures 1C and D).

*hackseq* demographics De-identified demographic data from *hackseq* participants in the pre-meeting survey/confirmation of attendance. Click here for additional data file. Copyright: © 2017 hackseq Organizing Committee 2016 2017 Data associated with the article are available under the terms of the Creative Commons Zero "No rights reserved" data waiver (CC0 1.0 Public domain dedication).

Post- *hackseq* survey responses De-identified post- *hackseq* survey response data for the figures. Click here for additional data file. Copyright: © 2017 hackseq Organizing Committee 2016 2017 Data associated with the article are available under the terms of the Creative Commons Zero "No rights reserved" data waiver (CC0 1.0 Public domain dedication).

## Technical and logistical requisites

Hackathons have little essential resource requirements. In this section, we outline the core logistics and technical infrastructure we employed. While these requisites could be stripped down, our experience was that attention and planning for these details maximized the efficiency of our teams to focus on coding and development.

### Core logistics

To encourage participation, *hackseq* had no entry-cost for participants. To ensure teams could focus on the hackathon and not technical or logistical issues, we secured funding for the venue, technical infrastructure, food, transportation and stationery by partnering with different organizations.

A sponsorship package was created to approach different academic, non -profit and industry organizations. Besides asking for financial support, we also made communication and marketing requests given that one of *hackseq’s* goals was to recruit a diverse pool of participants. A strong emphasis was placed on women’s groups in science and technology.

In November 2015, we contacted ASHG to ask if we could be a satellite event for their meeting. Given that ASHG 2016 conference was planned to be held in Vancouver, *hackseq* gained exposure from the ASHG's communication strategy. The ASHG also provided three travel grants to participants based on financial need and diversity.

These partnerships allowed *hackseq* to take place in a large, bright atrium at the University of British Columbia. This allowed all the teams to be in a single-space and interact with one another. Food was provided to minimize distraction and two social events were hosted, one the first night and one on the last night to encourage collaboration and networking amongst participants.

### Technical infrastructure

Reliable technical infrastructure is necessary for organizing a successful hackathon; primarily, electrical power, Internet access and computing resources. We ensured the venue had adequate electrical outlets for the participants’ laptops and organized a dedicated Wi-Fi network connection be established for the event through the university's information technology office.

Unlike many hackathons, *hackseq* was not restricted to coding. It also included genomic data analyses, which required additional computational resources. To promote reproducibility and collaboration, all the projects were based on pre-organized GitHub teams and repositories (see *hackseq* organization on Github; https://github.com/hackseq). To provide teams with reliable and powerful computation, we secured in-kind donation of cloud computing from Amazon Web Services Elastic Compute Cloud (AWS-EC2), and Canada’s Michael Smith Genome Science Centre genOmics Research Container Architecture (ORCA). We used Linuxbrew, a cross-platform package management tool, to install bioinformatics software on ORCA ^5^.

There was an equal usage of AWS-EC2 and ORCA amongst the participants (43%, not mutually exclusive) and an additional 12% using high-performance computing resources from their resident institutions. Users showed a preference for resources they were previously experienced with, and reported that it was not feasible to learn to use a new computing resource in the given time. Allowing team leaders and participants access to computing resources ahead of time in the future to ‘experiment’ and familiarize themselves with the different resources is advisable.

**Figure 2.**
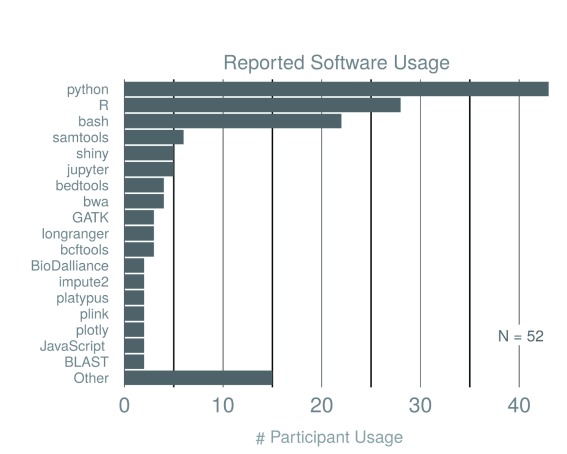
Software usage during hackseq 16. At the conclusion of hackseq, we asked participants to complete a survey on their experience at hackseq. There were 52 responses to the question, “Which programming languages and tools did you and your team use during the course of hackseq? (Comma delimited please).” These responses were parsed and the number of unique instances is reported. Languages or software listed <2 is reported as ‘Other’.

Each team chose which programming language and software they used. The majority of participants relied on Python (82.6%) and R (53.8%) programming languages and also used specialized software that related to their particular projects ( Figure 2).

In summary, the infrastructure requisites for running a successful hackathon are minimal and many can be acquired as in-kind donations from related organizations. In highlighting the essentials and key lessons, we hope to encourage the motivated reader to run a local scientific hackathon.

## Research project summaries

The projects undertaken during *hackseq* were from diverse fields within bioinformatics, ranging from human genomic variation analysis, microbial ecology and transcriptomics, to bioinformatic algorithm development. The projects were proposed by the team leaders, who defined the scope of the work, with the idea that at the end of the 72 hours there will have developed a working prototype. At *hackseq*, the teams organized organically, with team leaders defining the problem and teaching the participants the necessary background while participants offered their expertise on how to implement a solution. In this way a collaborative project goal could be explicitly defined (and re-defined), and the teams would work towards completing that goal together.

Here we provide brief summaries from the projects. Scientific abstracts, videos of final presentations and updated information on each project can be found at www.hackseq.com/projects16.

### VASCO: Visualization app for single cell exploration (led by Grace X.Y. Zheng)

Modern transcriptomics analysis tools have limited capacity for analyzing thousands of single-cell RNA-sequencing data (scRNA-seq). VASCO is an intuitive user-interface to visualize gene-cell expression and cell clustering data to explore the relationship between populations of cells and gene expression, including cell cluster of differentiation markers (CD-markers). This project was awarded the “People’s Choice” for the most outstanding project developed at *hackseq*.

### XYalign: Hacking sex chromosome variation (led by Melissa A. Wilson Sayres)

Human sex chromosomes violate typical ploidy assumptions made for NGS autosome copy number and variant measurement, which is further confounded by mis-alignment between the X and Y chromosomes. XYalign was developed to measure sex chromosome ploidy and remap reads based on the inferred sex for downstream analysis.

### ParetoParrot: A tool to optimize the parameters of command line software (led by Shaun Jackman)

Many bioinformatics software, such as genome sequence alignment and assembly, requires optimization of several input parameters to maximize a target metric. ParetoParrot measured the performance of several ‘black-box’ optimization algorithms to improve the performance of genome sequence assembly software.

### BaklavaWGS: Pseudo-WGS variant calling for common cell types aggregating ChIP-seq, RNA-seq and DHS from ENCODE and Roadmap Epigenomics data (led by Luca Pinello)

There is a wealth of sequencing datasets for cell types that have helped to understand and prioritize non-coding variants. Unfortunately, for many of those cell types we still don't have complete genotype information. BaklavaWGS recovers genotype data from cell lines aggregating sequencing data to aid downstream allele specific analysis. A preliminary analysis is available at http://www.baklavawgs.com/.

### Evaluating epigenetic modifications in ChIP-seq and methylation data across cell types and states (led by Manuel Belmadani)

A variety of datasets and approaches were investigated for analyzing cell type and state-specific genome regulation. The outcome of the experimental work in exploring differentially methylated regions from different epigenomic data and public databases, such as ENCODE ChIP-seq, IHEC and JASPAR, is presented.

### Selection of tag SNPs for an African SNP array by LD and haplotype based methods (led by Tommy Cartensen)

Commercial SNP arrays fail to capture the diversity of African populations and limit the capacity to conduct large-scale medical genetic studies. Using African whole genome sequencing (WGS) data, an algorithm was developed to quickly identify SNP tags for this population. This will be used to improve upon SNP arrays for this richly diverse continent.

### Somatic mutation from separated haplotypes (SMUSH) (led by Patrick Marks)

Calling somatic mutations relies on matched tumour and normal DNA sequencing, but a matched normal sample is often not available. The SMUSH algorithm was developed to differentiate wild type, germline and somatic mutations from linked-read DNA sequencing libraries.

### MetaGenius (led by Michael Schnall-Levin)

Analysis of shotgun metagenomic sequencing data is limited in its capacity to assemble over homologous sequences. MetaGenius uses linked-read DNA sequencing to improve the assembly of a mixture of five bacterial species.

### mICP: Metagenomic indicator contig predictor (led by Ben Busby)

Metagenomic sequencing has largely focused on 16S rRNA amplicons. This mICP strategy uses a mixture of long PacBio and short Illumina reads to identify contigs from environmental sequencing samples, which predict the environmental state from which they were found.

## Discussion

**Figure 3.**
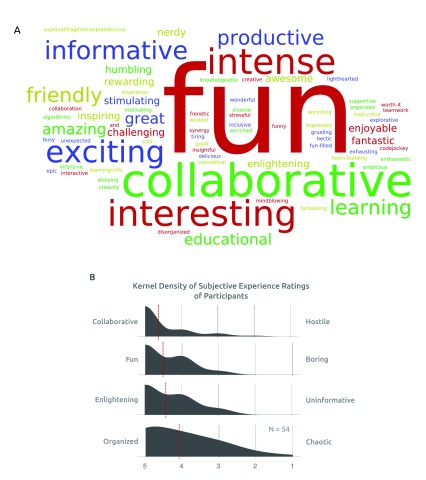
Quantification of subjective experience. To measure the quality of the experience *hackseq* participants had after the event, we asked ( **A**) “Please write three single word adjectives to describe your experience at hackseq?” Responses were parsed and used to make a word-cloud ( www.wordle.net), where the size of the word is proportional to the number of occurrences of that word in the survey responses. For scale, in 50 responses: ‘fun’ was mentioned 26 times; ‘exciting’ 6 times; and ‘supercalifragilisticexpialidocious’ once. ( **B**) Additionally, we asked participants to rate four dimensions of their experience on a linear scale from 1 to 5. The kernel density of responses for these dimensions are shown, with a red dotted line showing the mean value of the responses.

The overarching themes of *hackseq* were inclusivity, open science and collaboration. To gauge the extent to which we were successful in delivering on these themes, we performed a final survey at the conclusion of *hackseq*. Participants overwhelmingly described their experience as positive ( Figure 3), with 100% (n = 54) of the survey respondents indicating that they would participate in an event like *hackseq* again and a further 80% indicating that they would like to take on an organizational/leadership role in future *hackseq* events. Participants specifically highlighted that *hackseq* created ample recruitment, employment and collaborative opportunities, while also exposing participants to different datasets and analyses. We believe this reflects the underlying desire amongst young scientists to share, collaborate and learn from one another. They only need be given the opportunity to do so.

By organizing *hackseq* as a satellite meeting of an international conference like ASHG, we were able to attract team leaders and participants from around the world, including a large proportion of young investigators and female participants ( Figure 1). There was a higher proportion of females at *hackseq* (35.5%), than reported ratios at hackathons for which data is available, 20% at NASA’s Space Apps Challenge ( https://www.fastcompany.com/3059036/most-creative-people/what-do-women-want-at-hackathons-nasa-has-a-list) or 15% at Spotify-organized hackathons ( https://labs.spotify.com/2015/01/13/diversify-how-we-created-a-hackathon-with-50-50-female-male-participants/), which we believe to be a consequence of starting with a representative organizing committee and specifically encouraging female participation during recruitment. Although, this comparison is confounded by differences in starting demographics between computer science/engineering students and bioinformatics/biology students.

To further increase global representation at future *hackseq* events, we recommend providing additional targeted travel awards or remote participation options to reduce proximity/cost restrictions. Further improvements could include educational resources to address common technical issues, the provision of an overnight area for participants who would like to continue to work after hours and additional activities to encourage interaction with members from different teams.

## Conclusion

The nature of biological sciences has shifted to an increasing emphasis on computational analysis. Collaborative events, such as *hackseq,* offer an exciting platform to bring together a wide spectrum of scientists to work together and innovate. We present demographic information about the first *hackseq* hackathon and encourage future organizers to do likewise, to quantify social inequalities that may be present in such events, and strive to achieve equal representation in the sciences. It’s our hope that the information presented here will aid and encourage others in organizing genomics hackathons.

## Data availability

The data referenced by this article are under copyright with the following copyright statement: Copyright: © 2017 hackseq Organizing Committee 2016

Data associated with the article are available under the terms of the Creative Commons Zero "No rights reserved" data waiver (CC0 1.0 Public domain dedication). 

**Dataset 1: *hackseq* demographics:** De-identified demographic data from *hackseq* participants in the pre-meeting survey/confirmation of attendance. doi, 10.5256/f1000research.10964.d152802 ^6^

**Dataset 2: Post- *hackseq* survey responses:** De-identified post- *hackseq* survey response data for the figures. doi, 10.5256/f1000research.10964.d152803 ^7^
