# Supplementary material for: hackseq: Catalyzing collaboration between biological and computational scientists via hackathon
Source: F1000Res. 2017 Apr 10;6:197. Originally published 2017 Feb 28. [Version 2] doi: 10.12688/f1000research.10964.2 (PMC5381621; doi:10.12688/f1000research.10964.2)
Supplement: Peer review history

# Peer review: Referee response for version 2

**DOI:** 10.5256/f1000research.12239.r21700

**Published:** 2017-05-08

**Authors:** Kate L. Hertweck

**Affiliations:** 1 Department of Biology, The University of Texas at Tyler, Tyler, TX, USA

My comments have been accommodated.

I have read this submission. I believe that I have an appropriate level of expertise to confirm that it is of an acceptable scientific standard.

# Peer review: Referee response for version 2

**DOI:** 10.5256/f1000research.12239.r21699

**Published:** 2017-04-13

**Authors:** Jiarong Guo

**Affiliations:** 1 Center for Microbial Ecology, Michigan State University, East Lansing, MI, USA

My previous comments have been addressed by authors in the new version and also in comment section. I have no further comments to make.

I have read this submission. I believe that I have an appropriate level of expertise to confirm that it is of an acceptable scientific standard.

# Peer review: Referee response for version 1

**DOI:** 10.5256/f1000research.11818.r20964

**Published:** 2017-03-31

**Authors:** Jiarong Guo

**Affiliations:** 1 Center for Microbial Ecology, Michigan State University, East Lansing, MI, USA

The authors reported a detailed summary of their genomic hackathon, hackseq, to help those interested in organizing similar hackathons in future. The hackseq brought together 66 biological and computational scientists with diverse demographic background to collaborate on nine projects on genomics ranging from data visualization to algorithm development. All the participants had positive responses in post assessment and showed interests in future hackathons.

 The background is clearly articulated. There are detailed descriptions on hackseq format and technical and logistical requisites, which are useful for future hackathons. Brief research project summaries are also described with more information available on GitHub. The data for reproducing the figures are made available on F1000 and schedules and application forms are available on GitHub.

**Major comments:**

 Strengths:

 Overall, sharing details and experiences of the hackseq such as recruiting project leaders and participants, assigning teams, logistical and technical requisites, and post assessment is valuable for the open science community to organize future hackathons.

1. It is a great idea to organize the hackathon as a satellite event of bigger events. The bigger events can help on travel cost of participants and more importantly promote hackathon participations.
2. This hackseq is very successful at recruiting diverse participants, because it has a representative committee that encourages female, minority and early career scientist participations and also good promotion strategies that it partners with organizations such as Society for Canadian Women in Science.

Weakness:

 The team leaders seem to have a critical role in each project, but their roles and responsibilities during the hackathon are not clearly mentioned in the manuscripts.

**Minor comments:**

 Last paragraph in “Hackseq format” section: it states that participants ranked top three projects on which they would like to work, but there are four projects of choice in the application form.

 First paragraph in “Core logistics” section: stationary -> stationery.

 Second paragraph in “Discussion”: female percentage in biology is significantly higher than in engineering. Thus the direct comparison of female participation rate of hackseq to engineering (NASA and Spotify) hackathons is not meaningful.

 Discussion: assessment is difficult with participants from diverse background. Some discussion on the current assessment and possible improvement would be useful.

I have read this submission. I believe that I have an appropriate level of expertise to confirm that it is of an acceptable scientific standard.

# Peer review: Referee response for version 1

**DOI:** 10.5256/f1000research.11818.r20626

**Published:** 2017-03-31

**Authors:** Kate L. Hertweck

**Affiliations:** 1 Department of Biology, The University of Texas at Tyler, Tyler, TX, USA

Thank you to the authors for writing a summary of what seems to be a very successful collaborative coding event, with this manuscript in particular focused on preparation for the event, managing logistic concerns during the event, and an overview of the projects supported. The manuscript is quite well written, and I have no concerns about the content presented therein.

 I especially appreciate the recommendations for how to solicit diverse leaders/participants, engage with partner organizations, and carefully craft a sense of community among attendees. Moreover, the authors include suggestions on how to improve similar events in the future. The data reported here provide an important context for comparison for events which continue to encourage participation from underrepresented groups. 

 Although not highlighted in the paper, the itinerary for the three-day meeting described here includes a number of additional details which would be useful to other coding event organizers. For example, while the majority of meeting time was dedicated to team work, extra workshops and talks were offerred (e.g., introduction to git) that would be encourage skills development for students or other participants new to the field. I'll be interested to see whether this model for hosting a hackathon continues at ASHG or other meetings.

I have read this submission. I believe that I have an appropriate level of expertise to confirm that it is of an acceptable scientific standard.
